# Contemporary Use of Drug-Coated Balloons for Coronary Angioplasty: A Comprehensive Review

**DOI:** 10.3390/jcm13206243

**Published:** 2024-10-19

**Authors:** Nicola Verde, Giuseppe Ciliberti, Luca Pittorino, Marco Ferrone, Michele Franzese, Massimo Russo, Angelo Cioppa, Grigore Popusoi, Luigi Salemme, Tullio Tesorio, Giuseppe Di Gioia

**Affiliations:** 1 Division of Cardiology, Catheterization Laboratory, Montevergine Clinic, 83013 Mercogliano, Italy; nicoverde91@gmail.com (N.V.);; 2 Department of Advanced Biomedical Sciences, Federico II University, 80131 Naples, Italy; 3 Institute of Cardiology, Fondazione Policlinico Universitario A. Gemelli IRCSS, Catholic University of Sacred Heart, 00136 Rome, Italy; 4 Department of Clinical and Molecular Medicine, Sapienza University of Rome, Cardiology Division, Sant’Andrea University Hospital, 00189 Rome, Italy; 5 Division of Cardiology, Department of Systems Medicine, Tor Vergata University, 00133 Rome, Italy

**Keywords:** drug-coated balloon, coronary artery disease, paclitaxel-coated balloon, sirolimus-coated balloon

## Abstract

The interventional treatment of coronary artery disease (CAD) has undergone significant improvements thanks to technological innovations. Nowadays, percutaneous coronary intervention (PCI) with drug-eluting stent (DES) implantation is the standard of care for the treatment of CAD. Nevertheless, the non-negligible incidence of in-stent restenosis (ISR) and suboptimal results in various anatomical settings has led to the development of drug-coated balloons (DCBs). DCBs are catheter-based balloons whose surface is coated with an anti-proliferative drug (mainly Paclitaxel or Sirolimus) loaded onto the balloon surface with different technologies and dose concentrations. In the beginning, these devices were used for the treatment of ISR showing an excellent efficacy profile in the inhibition of intimal hyperplasia. Subsequently, several studies evaluated their use in other angiographical and clinical contexts such as de novo lesions, small vessel disease, diffuse coronary disease, bifurcation lesions, acute coronary syndromes, high-bleeding risk and diabetic patients. This comprehensive review aims to describe the main DCB platforms on the market, their fields of application with the main supporting studies and their future perspectives.

## 1. Introduction, Rationale, and Technical Considerations

Since Andreas Grüntzig and colleagues performed the first percutaneous coronary intervention (PCI) in 1977, the interventional treatment of coronary artery disease (CAD) dramatically changed [1].

The first angioplasties were performed using rudimentary balloons with a high rate of suboptimal results due to elastic recoil, acute vessel closure and negative vessel remodeling. The introduction of bare metal stents (BMS) partially solved these issues at the cost of a high rate of early in-stent restenosis (ISR) and stent thrombosis [2].

A few years later, the widespread adoption of dual antiplatelet therapy (DAPT) and drug-eluting stents (DES) lowered the rate of ISR and stent thrombosis, becoming the standard of care for PCI.

Nevertheless, even with the most recent stent platforms (second-generation DES), a non-negligible risk of ISR and late stent thrombosis persists [3].

In this context, drug-coated balloons (DCBs) are emerging as a promising alternative, both for the treatment of ISR and for the novo lesions. DCBs are catheter-based balloons that exhibit different levels of compliance and whose surface is coated with an anti-proliferative drug that inhibits intimal hyperplasia.

DCBs offer several potential advantages over DES: (1) Preservation of epicardial coronary vasoreactivity; (2) Avoidance of late stent thrombosis and allergy to metal or polymer; (3) Possibility of a future surgical revascularization; (4) A quicker reendothelization of the arterial wall with a potentially shorter duration of DAPT; (5) Treatment of special lesions’ and patients’ settings in which DES perform poorly (i.e., small vessels, long and narrow disease, diabetic and high bleeding risk patients).

Currently, two drugs can be loaded on the balloon’s surface: Paclitaxel and Sirolimus. The different technologies adopted by commercially available balloons will be reviewed in Section 2.

After balloon inflation, the coating drug is delivered on the vessel surface and permeates through the vessel wall, where it exerts its inhibitory effects on the migration of smooth muscle cells and fibroblasts from media to intima, on their proliferation and on the secretion of the extracellular matrix.

An optimal lesion preparation is pivotal to successfully use DCBs: pre-dilatation should be performed using semi-compliant or non-compliant balloons with a 1:1 balloon-reference vessel diameter ratio. This creates an interruption in the intima continuity that facilitates the drug’s spread. If the lesion cannot be adequately prepared with traditional balloons due to a high calcific burden, devices such as high-pressure non-compliant balloons, cutting- and scoring-balloons, atherectomy devices (rotational and orbital atherectomy, laser atherectomy), or intravascular lithotripsy could be used. In the treatment of ISR, aggressive pre-dilatation is especially recommended when dealing with stents under expansion.

A good lesion preparation is evaluated as follows: (1) residual stenosis < 30%; (2) balloon-vessel reference diameter ratio of 1:1; (3) TIMI (Thrombolysis In Myocardial Infarction) flow grade 3; (4) absence of flow-limiting dissection (dissection type A and B are acceptable) [4]. Some studies focused their attention on intravascular imaging-guided lesion preparation (with intravascular ultrasound or optical coherence tomography). The meticulous and multiple pre-dilatation in de novo lesions, assisted by imaging methods and compared with traditional angiography, showed a significant gain in luminal area and in dissection angle, both predictors of better long-term angiographical outcomes (assessed mainly as late lumen loss and residual diameter stenosis at the angiographic follow-up) [5,6]. However, definite criteria to define an acceptable or optimal result are still lacking.

Accurate lesion preparation is also important because DCBs are generally less deliverable than standard balloons due to a larger crossing profile. This problem could particularly arise in extreme tortuosity, severe calcification, or distal lesions with the possibility of delivery failure.

The handling of DCBs should be performed with care, avoiding directly touching the balloon. Once the cover is removed, DCBs should be positioned as quickly as possible to avoid excessive drug loss from the balloon surface. Generally, the maximum transit time is provided by each manufacturer. Moreover, most companies do not recommend the reinsertion of the same DCB after a delivery failure or its reuse after the treatment of a single lesion. The minimum inflation time varies from 30 to 120″ and is specified by the manufacturer. The DCB should be at least 4 mm longer than the lesion to be treated in order to go beyond the lesion 2 mm distally and proximally.

## 2. Design and Technology of DCBs

Local drugs delivered for vascular treatment during PCI have been investigated for more than 30 years. The first experiments in vitro and in animal models showed that the local administration in a liquid solution of Paclitaxel, an anti-tumoral agent, led to an effective inhibition of neointimal proliferation [7]. This effect is possible due to the long persistence of the drug in the arterial wall allowed by specific hydrophobic binding sites in the arterial tissue [8]. Since Paclitaxel has low solubility in aqueous media, it soon became clear that a hydrophilic excipient could improve the release kinetics, thus reducing drug loss related to blood flow [9].

Therefore, an X-ray contrast agent, Iopromide, was initially identified as an excellent excipient both when Paclitaxel was directly injected into a coronary artery after BMS implantation and when an angioplasty catheter balloon was coated with Iopromide and Paclitaxel to prevent neointimal hyperplasia [10].

In addition to excipients, another fundamental component of the DCBs is the coating polymer, whose function is to hold the drug and to control its release. These coatings are made of bioabsorbable, biocompatible, and biodegradable substances with an adhesive nature. When the balloon is inflated, the polymeric coating adheres to the endothelium reducing the risk of thrombosis and holding the drug on site, facilitating its release and tissue absorption [11,12,13].

Over the years, numerous studies have investigated the coating procedure, optimal balloon contact-to-vessel time, drug persistence in the arterial wall, optimal drug concentration on balloon surface, alternative coating formulations and alternative antiproliferative drugs.

More recently, the safety and effectiveness of Limus drugs used on DES pushed the industry to develop DCBs with Sirolimus and other mTor inhibitors.

Several different DCBs based on different technologies are commercially available (Table 1). In this chapter, we will review the most commonly used device to date.

### 2.1. Paclitaxel-Coated Balloons

Paclitaxel is extracted from the bark of the Pacific Yew tree (*Taxus brevifolia*). It remains the most studied anti-proliferative agent and is still today the drug of choice for DCBs with a dose ranging between 2 and 3.5 μg/mm^2^. Paclitaxel hinders cellular proliferation by impeding the disassembly of microtubules, a component of cellular cytoskeleton, during mitosis. This action arrests cells in the G2/M phase of the cell cycle, making Paclitaxel a cytotoxic drug used as an anti-tumoral agent in the treatment of various types of cancer.

For this reason, there was concern regarding the use of Paclitaxel-coated balloons. Various safety pre-clinical studies demonstrated that Paclitaxel inhibits smooth muscle cell migration and proliferation at very low concentrations [7,14].

For cancer treatment, the typical systemic dose is around 300 mg, while on a Paclitaxel-coronary balloon of 3.0 × 20 mm and a concentration of 3 μg/mm^2^ there is about 0.4 mg of the drug. This dose is significantly below the minimal toxic dose. The dose of eluted Paclitaxel was shown to also be safe in the femoral-popliteal district, in which the balloons are bigger, with a higher amount of drug coated on the balloon surface, and the contact times being longer than for CAD treatment [15].

The first Paclitaxel DCB was the **Paccocath** (Bavaria Medical Technology, Webling, Germany) developed by Scheller and Speck and experimented on humans for the first time in 2003. In this context, 3 μg/mm^2^ of Paclitaxel is incorporated into a bioabsorbable matrix containing iopromide spacers. The Paccocath balloon was studied for the first time in the setting of ISR of BMS. In the ISR I and II trial, patients with ISR treated with Paccocath DCB compared with a conventional uncoated balloon had significantly better angiographic results in terms of late luminal loss and similar clinical outcomes at the 12-month follow-up (FU) [16,17].

The **SeQuent Please** (B Braun Vascular Systems, Melsungen, Germany) is an upgrade of Paccocath technology. Incorporating Paclitaxel into a specifically engineered bioabsorbable matrix results in an ameliorated release profile of the drug during PCI. A small amount of Iopromide as an excipient improves the solubility of Paclitaxel with a significant amount of the drug (approximately 80%) released during balloon inflation [8].

The **AGENT** (Boston Scientific, Marloborough, MA, USA) is the first and only DCB, to date, to recently receive FDA approval for the treatment of ISR in the United States. The AGENT is a semi-compliant balloon coated with a low dose formulation of Paclitaxel (2 μg/mm^2^, a dose lower respect its competitors) and uses a proprietary coating technology (TransPax) consisting of a uniform crystalline formulation of Paclitaxel and acetyl tributyl citrate (ATBC) as the excipient [18]. Despite the lower Paclitaxel density on the surface of the balloon, preclinical studies showed a similar vascular response compared with higher concentration Paclitaxel dose DCBs and similar tissue levels [19].

The **DIOR** (Eurocor GmbH, Bonn, Germany) is a paclitaxel-coated coronary balloon on the market for the treatment of de novo lesions, ISR and bifurcation lesions [20]. The first generation of this device incorporated microcrystals of Paclitaxel dissolved in dimethyl sulfoxide, which were coated onto a nano-porous balloon surface. Within the first 30 s of inflation, only 20% of the drug was released from the balloon surface [21].

To address this issue, a second DIOR generation was developed employing Shellac, a natural resin consisting of shellolic and aleuritic acid, as an additive. In this process, Paclitaxel is dissolved in shellac with a 1:1 ratio and applied as a coating onto the micro-porous surface structure of the DIOR balloon. Upon contact with bodily fluids, the hydrophilic shellac undergoes swelling, thereby opening the structure and facilitating the rapid release of Paclitaxel under pressure from the inflated balloon [22].

With this technology, a 30–45 s inflation time permits a 2- to 20-fold increase in drug availability at the tissue level. Additionally, the drug is uniformly distributed, swiftly delivered and effectively penetrates both horizontal and vertical dissections.

The **IN.PACT** (Medtronic Vascular Inc., Minneapolis, MN, USA) is a DCB technology based on Paclitaxel contained within a hydrophilic matrix together with urea, which facilitates Paclitaxel release with a loading dose on the surface of the balloon of 3.5 μg/mm^2^ [23]. There are three commercially available types of IN.PACT balloons (IN.PACT Admiral for peripheral artery disease, IN.PACT Falcon for CAD treatment and IN.PACT Amphirion for below-the-knee disease).

The **Elutax “3”** balloon (AR Baltic Medical, Vilnius, Lithuania) is a third-generation Paclitaxel DCB designed to be used in different vascular territories: coronary artery disease, intra-cranial and carotid lesions, peripheral artery disease, artero-venous fistula, large arteries and veins. Elutax “3” has a modified drug-coated surface, incorporating a three-dimensional dextran–paclitaxel formation with a recommended inflation balloon time of 30 s and a concentrated Paclitaxel dose of 2.2 μg/mm^2^.

**Pantera LUX** (BIOTRONIK AG, Berlin, Germany) is a DCB utilizing butyryl-trihexyl citrate (BTHC) loaded with Paclitaxel at a dosage of 3 μg/mm^2^ specifically designed for small vessels and de novo lesions but also for ISR treatment. BTHC is a rapidly metabolized, safe, and biocompatible excipient allowing for excellent bioavailability at the target site with detection in coronary porcine models of the drug beyond 7 days after one application [24].

The **Restore** Paclitaxel-coated balloon (Cardionovum, Bonn, Germany) is a Paclitaxel-coated balloon with a concentration dose of 3 μg/mm^2^ and an innovative SAFEPAX shellac-ammonium salt excipient, which might avoid downstream effects to minimize the potential risk of microembolization and wash-off phenomenon during catheter delivery. In some studies, it showed its efficacy and safety profile for ISR and small vessel disease treatment [25,26].

### 2.2. Limus-Coated Balloons

In more recent years, Limus drugs, and particularly Sirolimus, a lipophilic drug, have undergone examination for their potential application in DCBs. Sirolimus (rapamycin), a macrocyclic lactone, is derived from a strain of Streptomyces hygroscopicus, originally isolated from a soil sample obtained from Rapa Nui, more commonly recognized as Easter Island. While initially identified for its antifungal properties, further investigations unveiled notable antitumor and immunosuppressive activities associated with Sirolimus [27]. Sirolimus engages in the formation of an immunosuppressive complex with the intracellular protein FKBP12. This complex effectively inhibits the activation of the cell-cycle-specific kinase, mTOR. Subsequent downstream events following mTOR inactivation led to the obstruction of cell-cycle progression at the junction of the G1 and the S phase, thereby limiting the migration and proliferation of smooth muscle cells. The main limitation in the use of Sirolimus in DCBs is attributed to its lower tissue transfer and penetration capacity compared to Paclitaxel, along with the requirement to remain in place for an extended duration due to its reversible binding with mTOR. To overcome these limitations, various solutions have been developed to implement Sirolimus DCBs.

**MagicTouch** (Concept Inc., Gujarat, India) is a Sirolimus DCB in which nanoparticles composed of Sirolimus encapsulated within a phospholipid layer are sprayed on the balloon surface to obtain a uniform coating with a drug concentration of 1.27 μg/mm^2^. The purpose of this technology is to achieve rapid drug release, reducing loss of drug during delivery, and increasing the bioavailability and biocompatibility.

**SELUTION** (Med Alliance, Nyon, Switzerland) is a Sirolimus DCB in which a biodegradable polymer (Poly-lactic-co-glycolic acid–PLGA) encapsulates Sirolimus into micro-reservoirs that control drug release through matrix degradation [28]. The drug coating is homogeneous across the balloon’s surface, featuring a Sirolimus concentration of 1 μg/mm^2^. The drug exhibits high retention on the balloon during both preparation and delivery, and its elution profile closely resembles that of currently available Limus based DES.

Another currently available Sirolimus DCB is the **SeQuent Please SCB** (B Braun, Melsungen, Germany). It uses a Sirolimus coating in crystalline form with an additive of butylated hydroxytoluene (BHT) at a dose of 4.00 μg/mm^2^ (a significantly higher dose than the previous ones). This formulation allows a prolonged retention of Sirolimus in the vessel wall with 40 to 50% amount of the drug found in the vessel wall after 1 month [29].

The **Mozec SEB** (Meril, Gujarat, India) is a recently commercialized Sirolimus-eluting balloon with a formulation of solid lipid nanospheres consisting of Sirolimus and lipid particles < 500 nm. The lipid nanoparticles have a high tissue diffusion coefficient with prolonged tissue residence time (the formulation ensures drug availability for 1 month). The drug concentration for this device is of 3 μg/mm^2^ [30].

Another derivative of Sirolimus is **Biolimus A9**, a proprietary formulation (Biosensors International, Singapore, Singapore), was used to develop a novel DCB. This molecule is 10-fold more lipophilic than Sirolimus, maintaining the same mechanism of action. The Biolimus A9 drug-coated balloon is a semi-compliant balloon with a drug concentration of 3 μg/mm^2^ and polyethylene oxide as the delivery matrix. Pre-clinical testing on the coronary porcine model demonstrated the effective permanence of Biolimus up to 28 days after the procedure [31]. Recently, this device showed comparable angiographical outcomes in the treatment of DES-ISR compared with a CE-approved Paclitaxel DCB at a 9-month follow-up [32].

**Figure 1:**
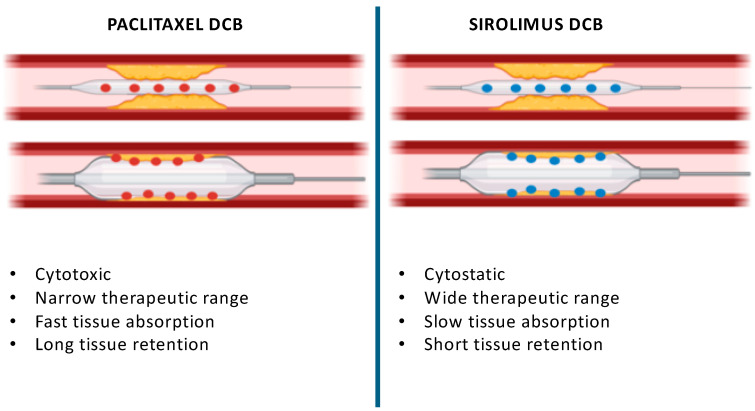
Main differences between Paclitaxel and Sirolimus DCBs.

Given the remarkable differences among the different DCBs in terms of the drugs used, formulation, release kinetics, and permanence of the drug within the arterial tissue, it is a common opinion that there is no “class effect” for either Paclitaxel- or Sirolimus-coated balloons. Figure 1 depicts the main differences between Paclitaxel and Limus DCB.

## 3. Clinical Settings

Currently, the use of coronary DCBs is primarily recommended in the treatment of ISR [33]. The application of DCBs is rapidly expanding into novel areas, including the treatment of native small vessels (diameter < 2.75–3.0 mm), bifurcation lesions, chronic total occlusion (CTO) or in specific clinical settings like diabetics or high bleeding risk (HBR) patients.

In this chapter, we will summarize the main contexts for the use of DCBs along with the corresponding supporting studies.

### 3.1. In-Stent Restenosis

In-stent restenosis is generally defined as a significant reduction in luminal diameter at the site of a previously implanted stent (≥50% within the stented segment or 5 mm proximal or distal to the stent) after a successful PCI and is considered the main cause of stent failure, ranging from 5 to 10% of all PCI procedures [34]. It is important to underline that BMS-ISR and DES-ISR should be considered as different pathological entities: the BMS-ISR has typically a diffuse pattern, characterized by excessive neointimal hyperplasia, and tends to develop more rapidly, whereas the DES-ISR is generally caused by focal late neoatherosclerosis and might be perceived as more complicated because of antiproliferative drugs’ insufficient effect [35].

Although PCI with DES remarkably decreased the incidence of ISR compared with BMS, ISR remains a relevant problem, especially in patients with a high burden of comorbidities [36]. In this scenario, the role of DCB therapy for ISR lesions has been studied extensively and has been appointed a class 1A recommendation according to the European Society of Cardiology (ESC) guidelines in 2018 [33]. Overall, DCB in patients with ISR was shown to have similar efficacy and sometimes be superior to BMS and/or DES in terms of angiographic and clinical outcomes. Table 2 summarizes randomized controlled trials comparing DCB and BMS/DES for ISR [16,17,37,38,39,40,41,42,43,44,45,46,47,48].

In the setting of BMS-ISR, PACCOCATH ISR-I and -II [16,17] were the first clinical trials to show that paclitaxel-coated balloons (PCBs) significantly decreased late lumen loss (LLL) and major adverse cardiovascular events (MACE) compared to “plain old balloon angioplasty” (POBA). In line with previous results, PEPCAD II and TIS trials showed a significant advantage in terms of LLL reduction also compared with paclitaxel-coated DES (PES) and everolimus-coated DES (EES), respectively [37,40].

In the setting of DES-ISR, clinical trials proved the advantage of DCB on LLL, diameter stenosis (DS), minimum lumen diameter (MLD), binary restenosis (BR), and target lesion revascularization (TLR) compared with PES or EES [41,42,43,44,45], without a significant reduction in MACE. Moreover, the PEPCAD-DES trial showed that PCB was superior to POBA alone in reducing MACE for the treatment of DES restenosis [46].

The effects of PCB drug concentration in this setting were recently investigated. The results from the 7 years follow-up of the AGENT (low-dose formulation PCB) as compared to the SeQuent PCB (high-dose formulation) for the treatment of DES-ISR showed similar outcomes between the two groups in terms of death, target lesion revascularization/thrombosis and MI [49].

On the same line, a meta-analysis including six studies involving 1322 patients comparing ISR treatment (regardless of BMS- or DES-ISR) with DCB or POBA showed clear superiority of DCBs both in terms of angiographical (TLR, 0.22 OR; 95% CI 0.10 to 0.47; *p* = 0.0001) and clinical outcomes (MACE, 0.14 OR, 95% CI 0.08 to 0.25; *p* < 0.00001) [50].

Despite the relevant results of DCB in the management of ISR, some meta-analyses of randomized controlled data comparing PCI with DES and PCB have shown that repeat DES implantation is moderately more effective in increasing MLD and reducing TLR rate, residual DS and the need for subsequent TLR [51,52]. A network meta-analysis showed that PCI with EES was the most effective treatment, while DCBs were ranked as the second most effective ISR treatment, without significant differences compared to Sirolimus-eluting stents (SES) or PES. These findings suggested that both strategies may be considered for ISR therapy: DES for the best angiographic and clinical outcomes, and DCB for providing favorable results without adding a new stent layer [53]. Nevertheless, the 2024 ESC guidelines on chronic coronary syndromes state that both DES and DCBs can be used for ISR treatment, with a preference for DES (Class IA) [54]. It remains unclear whether concomitant factors such as age, frailty risk, history of heart failure, obesity, and CKD, may play a role in modulating end-points in the DCB and DES groups.

Future evidence will emphasize the importance of selecting the type of DCB based on the clinical and angiographic setting: to date, while SCB failed to demonstrate non-inferiority for angiographic net lumen gain compared to PCB for the treatment of de novo small vessel disease [55], some clinical studies showed that Sirolimus lead to better clinical and angiographical results compared with Paclitaxel balloons in patients with DES-ISR, although the differences were not statistically different [56,57]. Furthermore, another DCB has recently shown efficacy in the treatment of DES-ISR: the Biolimus A9 DCB (BCB), which has shown similar angiographic results in the treatment of coronary DES-ISR compared to a clinically tested PCB [32]. Further data are needed in this regard.

### 3.2. De Novo Lesions

#### 3.2.1. De Novo Lesions in Small Vessels

Coronary small-vessel disease, generally defined as lesions in vessels < 2.75 or <3.0 mm, still remains a challenge in the daily practice of interventional cardiologists. Although DESs are effective in both small and large vessels, the resulting late lumen loss (LLL), defined as the difference between the post-procedural vessel diameter and the vessel diameter at follow-up, occupies a higher percentage of the respective vessel diameter, leading to higher rates of ISR and clinical events [58,59]. After an angioplasty, the minimum lumen diameter (MLD) initially enlarges and subsequently decreases, causing a LLL, primarily due to neointimal proliferation or hyperplasia [60]. For these reasons, multiple RCTs have explored the effectiveness of DCBs compared with POBA, BMS and DES in the treatment of SVD [61].

The PICCOLETO Trial was the first randomized trial that compared an early-generation PCB (DIOR I) with PES. The primary study endpoint was non-inferiority for percent diameter stenosis at 6-month angiographic follow-up; secondary endpoints were angiographic binary restenosis and occurrence of MACEs. The study was prematurely interrupted because of better results in the PES arm (increase in MACE and in target vessel restenosis in the PCB group). However, this study had some limitations: the low efficacy of the early generation PCB selected with a lower concentration of drug at tissue level and the low rate of pre-dilatation in lesion preparation (around 25% of total) [62].

The BELLO trial evaluated non-inferiority for the Falcon PCB vs. the Taxus Liberté PES. The primary endpoint was non-inferiority for angiographic in-stent (in-balloon) late loss. Secondary endpoints were restenosis and TLR at angiographic follow-up and MACE. Late angiographic findings were similar in the two arms although the LLL was significantly reduced in the PCB arm. In this trial, bail out stenting was required in 20% of patients in the PCB arm. During the 6-month and 3-year follow-up, the incidence of TLR and MACEs in the PCB group was lower than in the PES group [63].

The first trial that compared a paclitaxel-iopromide DCB with a second-generation DES was the BASKETSMALL 2 trial. The primary outcome was MACE. At 12-month follow-up, in a full-analysis population, the proportions of MACE were similar in both study groups (7·3% for the DES group vs. 7.5% for the DCB group; hazard ratio [HR] 0.97 [95% CI 0.58–1.64], *p* = 0.9180). After 3 years, the same rate of MACE was reported in both groups (15%, hazard ratio [HR] 0.99, 95% CI 0.68–1.45; *p* = 0.95). Nonetheless, the incidence of vessel or stent thrombosis and serious bleeding in the PCB group was lower than in the DES group, with a more pronounced benefit in diabetic patients [64].

In the RESTORE SVD trial, patients were randomized to PCI with PCB or zotarolimus-eluting stent (Resolute Integrity). The main endpoint was in-segment percentage diameter stenosis at 9 months. Similar in-segment percent diameter stenosis was found and there was no difference in TLR, cardiac death, myocardial infarction and any revascularization. No difference was also found in target lesion failure after 1 year and up to 2 years [65]. These findings may support the use of DCB as an alternative to DES in SVD but take into account meticulous lesion preparation.

The PICCOLETO II trial was a randomized non-inferiority study comparing PCB (Elutax) with EES (Xience) in small vessel disease. The primary study endpoint was in-lesion LLL at 6 months. Secondary endpoints were minimal lumen diameter, percent diameter stenosis at angiographic FU, and the occurrence of MACE at 12 months. No significant differences were found in MACE, spontaneous MI and vessel thrombosis but LLLs were significantly lower in the DCB group [66].

In summary, the existing evidence indicates that DCBs are effective and safe for treating small vessel lesions, with a reduced incidence of restenosis and comparable or superior clinical outcomes compared to stents. TRANSFORM II trial is currently ongoing multicenter, noninferiority, RCT evaluating an SCB against the standard of care for native coronary vessels with a 2–3 mm diameter, in terms of 12-month target lesion failure (TLF; primary endpoint) and MACE (coprimary endpoint) [67].

#### 3.2.2. De Novo Lesions in Large Vessels

The role of DCBs in de novo lesions in the treatment of large coronary vessels (≥3.0 mm) is appealing, offering the advantage of avoiding stent-struts under-expansion and malapposition, especially in some settings like calcified lesions or bifurcations. Intraprocedural dissection is more common in PCI performed with DCB, but there is some evidence suggesting that non-flow-limiting dissection does not automatically lead to worse clinical outcomes [68]. In an Italian prospective observational study, among 156 patients who received DCB PCI, 52 were left with an angiographically visible dissection. At 6 to 9 months, no more dissections were observed in 45 patients. At the 9-month follow-up, no significant increase in MACE was observed in patients left with a dissection [69].

The DEBUT trial compared the use of drug-coated balloons (DCBs) to bare-metal stents (BMS) in treating de-novo coronary artery lesions in patients with a high risk of bleeding. It was a single-blind, randomized, non-inferiority trial conducted across five sites in Finland. The primary outcome was the occurrence of MACE at 9 months, which occurred in 1% of patients in the DCB group and 14% of patients in the BMS group (absolute risk difference −13.2% [95% CI −6.2 to −21.1], RR 0.07 [95% CI 0.01 to 0.52]; *p* < 0.00001 for non-inferiority and *p* = 0.00034 for superiority) [69].

In a prospective, real-world all-comers registry conducted from December 2013 to December 2015, 757 patients with coronary lesions were treated with the IN.PACT Falcon balloon. The primary outcome assessed was the clinically driven TLR rate at 12 months. Among 805 lesions, 43.1% were de novo, and 53.2% were ISR (DES or BMS). TLR at 12 months was 6.2% and TVR 8.3%. MACE were recorded in 9.7% of patients, comprising 0.8% for cardiac death and 2.7% for myocardial infarction, along with TLR/TVR. Subgroup analysis revealed that the TLR rate was 7.5% for ISR (2.1% BMS and 9.5% DES) and 4.9% for de novo lesions [70].

Yu et al. conducted a clinical trial in which 527 patients (595 lesions) underwent PCI with a DCB (SeQuent Please^®^) [71]. Two hundred and twenty-two of these lesions involved large vessels (considering 2.8 mm as cut-off diameter). During the FU, there was a lower incidence of MACE and TLR in the large vessel group than in the small vessel group [71].

Also, Shin et al. conducted a study to evaluate the effectiveness of PCB and DES in treating large vessel lesions (diameter between 2.5 mm and 3.5 mm). The conclusive data showed lower LLL in patients under DAPT treated with DCB rather than DES (0.05 ± 0.27 mm vs. 0.40 ± 0.54 mm, *p* = 0.03) [72].

In the international real-world DELUX registry, 1064 patients with 1137 lesions were enrolled at 62 sites in 12 countries. Nine hundred and eighteen patients (86.3%) presented with ISR (499 [54.4%] with BMS-ISR, 419 [45.6%] with DES-ISR), 105 patients (9.9%) presented with de novo lesions, and 41 (3.8%) could not be definitively classified into a single group due to the presence of multiple lesion characteristics per patient or the unavailability of specific information.

The study endpoint was MACE, defined as a composite of all-cause mortality, non-fatal myocardial infarction and clinically driven target vessel revascularization, and was 7.0% in the de novo population at six months and 9,4% at 12 months. At 12 months, there was a trend toward higher MACE rate (15.7% vs. 9.4%, *p* = 0.10) and significantly higher TVR rates (10.2% vs. 3.1%, *p* = 0.03) in patients with ISR rather than de novo lesions [73].

Recently, Colombo et al. evaluated a DCB-based treatment on the left anterior descending artery in 147 patients (43 patients with DCB only, 104 with a hybrid approach) and compared them to 701 patients who received a DES-based treatment. Endpoints were defined according to the Academic Research Consortium-2 criteria. The primary endpoint was the incidence of TLF, defined as the composite of TLR, target vessel myocardial infarction and cardiac death at 2 years. Secondary endpoints encompassed the individual components of the primary endpoint. This includes target vessel failure, defined as the composite outcome of cardiac death, TVR and target vessel myocardial infarction, as well as all-cause mortality, target lesion thrombosis, and non-TVR events. No significant difference was observed in the cumulative 2-year TLF incidence between the two groups. The total treated length was greater in the DCB group (65 [40,41,42,43,44,45,46,47,48,49,50,51,52,53,54,55,56,57,58,59,60,61,62,63,64,65,66,67,68,69,70,71,72,73,74,75,76,77,78,79,80,81,82] mm) compared to the DES group (56 [46,47,48,49,50,51,52,53,54,55,56,57,58,59,60,61,62,63,64,65,66] mm; *p* = 0.002). Additionally, longer DES were utilized (38 [24,25,26,27,28,29,30,31,32,33,34,35,36,37,38,39,40,41,42,43,44,45,46,47,48,49,50,51,52,53,54,55,56,57,58,59,60,61,62] mm vs. 56 [46,47,48,49,50,51,52,53,54,55,56,57,58,59,60,61,62,63,64,65,66] mm; *p* < 0.001), and a larger percentage of significant vessels were treated in the DES cohort (76.2% vs. 83.5%; *p* = 0.036). The 2-year cumulative incidence of TLF did not significantly differ between the two cohorts (4.1% in the DCB group vs. 9.8% in the DES group; hazard ratio 0.51 [95% CI, 0.20–1.27]; *p* = 0.15). Following a 1:1 propensity score matching that produced 139 matched pairs, DCB-based treatment was associated with a lower risk for TLF at 2 years compared with DES-only PCI (hazard ratio 0.2 [95% CI, 0.07–0.58]; *p* = 0.003), primarily due to the reduced need for TLR [74].

Although recent studies have shown positive therapeutic effects, further studies are needed to confirm the benefits of angioplasty with DCBs in large vessels.

### 3.3. Diffuse Coronary Disease

Treating diffuse CAD with DCBs is an emerging approach, especially in cases where traditional stenting may not be practical due to the extensive nature of the disease. Long lesions often require stent implantation with a long overlap, which leads to an increased incidence of undesirable consequences such as stent thrombosis and restenosis. Also, a “full metal jacket” in young patients would preclude the possibility of future surgical options. A strategy integrating the use of DES (proximally) and DCB (distally) was suggested for de novo diffuse long lesions treatment (hybrid approach). A recent trial explored the effectiveness of using DCB alone or combined with DES to treat diffuse de novo lesions exceeding 25 mm. The study revealed that both strategies had similar outcomes in terms of MACE and TLR. Among the DCB-treated lesions, 56.0% were treated solely with DCB, 36.6% with a hybrid approach, and 7.4% with DCB and DES as a bail-out. At a 2-year FU, outcome rates for DCB ± DES were similar to those for DCB alone (MACE = 20.8% vs. 22.7%, *p* = 0.74; TVR = 14.8% vs. 11.5%, *p* = 0.44; TLR = 9.6% vs. 9.3%, *p* = 0.84) [75].

In a prospective, observational, multicenter study, patients with diffuse coronary lesions (>25 mm) underwent DCB and/or DES. The DCB cohort included 355 patients (360 lesions), of these 142 patients (143 lesions) received the DCB-only strategy while 213 patients (217 lesions, 60.3%) received the hybrid strategy. A total of 672 patients (831 lesions) were managed with DES alone. The primary outcome of interest was TLR over a 3-year FU, while the secondary outcome was MACE, encompassing all-cause mortality, non-fatal myocardial infarction, and TVR. Over the 3-year FU period, no significant differences in TLR or MACE were detected. Additionally, no cases of thrombosis were reported in the DCB group, whereas four instances of stent thrombosis occurred in the DES group. Comparable rates of TLR and MACE were observed between the DCB-only and hybrid strategies [76].

The HYPER pilot trial will assess the 12-month clinical results of a hybrid approach treatment in 100 patients with diffuse coronary lesions [77].

### 3.4. Bifurcation Lesions

Coronary bifurcation lesions are treated in 15–20% of all PCI cases and still represent a challenging scenario [78]. According to the European Bifurcation Club consensus, for most cases, the recommended approach is the provisional strategy, namely placing a stent in the main branch (MB), with side branch (SB) balloon dilatation or provisional stenting as needed [33]. Indeed, routine double-stent placement does not notably enhance the prognosis and may even lead to higher rates of hospitalization and MACE [79]. Nevertheless, placing a single stent can change the bifurcation’s structure and harm the SB, potentially restricting collateral flow and causing myocardial ischemia or even occlusion [80]. One of the strategies to open the stent struts towards the SB and to ameliorate flow is to perform plain old balloon angioplasty (POBA). However, SB-POBA in the context of MB-stenting is associated with a high rate of restenosis [81]. DCB might represent a potential alternative to POBA and/or DES for SB-PCI: it maintains the bifurcation’s natural structure, particularly crucial in the carina area ensuring even distribution of high-dose antiproliferative drugs across the entire blood vessel surface [82,83]. In this scenario, sequential DCB dilatation following optimal lesion preparation (i.e., pre-dilatation) is crucial for maximizing drug transfer and bioavailability [84]. The suboptimal result after pre-dilatation before DCB (TIMI flow < 3, DS > 30% and major dissections) is an independent predictor of TLR [80]. Instead, non-flow-limiting dissections undergo self-healing in more than 90% of cases and do not significantly impact clinical outcomes [85].

To date, studies on SB-PCI with DCB have reported good results in terms of angiographic and clinical outcomes. Table 3 shows randomized controlled trials on DCB for bifurcation lesions [86,87,88,89].

In lesions without proximal MB involvement, a strategy of SB-DCB significantly reduced LLL and restenosis rate compared to POBA [83]. In studies designed with DES/BMS PCI in the MB, the use of DCB in the SB brought benefits in terms of LLL, rate restenosis and TLR, without any significant repercussions on the long-term cardiac events [86,87,88]. A recent meta-analysis by Zheng et al. included 10 studies with a total of 934 patients that also showed the superiority of DCB over POBA in SB protection for de novo coronary bifurcation lesions [90]. However, the latest consensus from the European Bifurcation Club (EBC) advises against the routine use of DCB for de novo bifurcation lesions. This recommendation is due to the lack of strong evidence and the absence of comparative studies between DCB and DES for treating bifurcation SB [91].

### 3.5. Chronic Total Occlusions

Chronic total occlusion (CTO) is defined as a completely occluded coronary artery without any antegrade flow for a duration of at least 3 months. Although CTO patients often have good collateral circulation, they may have ischemic areas and experience anginal symptoms and MACE [92]. Therefore, CTO recanalization should be attempted in the presence of angina despite medical treatment or reversible myocardial ischemia (level of evidence IIA/B according to the 2018 ESC/EACS guidelines for myocardial revascularization) [34]. However, CTO lesions are typically hard and calcific, and this may contribute to stent under-expansion and malapposition, increasing the risk of stent failure. In addition, the size of totally occluded vessels is often underestimated leading to the implantation of smaller stents. In fact, the use of BMS/DES in CTO lesions was linked to a higher rate of cardiac events as compared to non-CTO lesions [93]. Although the efficacy of DCB in such lesions has not been extensively investigated, it may be considered as a reasonable option if the stent is not expected to be adequately expanded [94]. Furthermore, patients with CTO often have a relevant burden of comorbidities that place them at high bleeding risk [95]. Therefore, the use of DCB might shorten the implanted metal, reducing in-stent restenosis/thrombosis and the duration of antiplatelet therapy. A German multicenter study was the first to show the applicability of DCB in the treatment of CTO, highlighting a high rate of recanalization and a low rate of restenosis [96]. Encouraging results also emerged from a Korean study that highlighted how the LLL in the DCB group was lower than in the DES group [97].

Given that patients with CTO frequently present with multiple lesions, and previous research has established a link between the number/length of stents and adverse events, some investigations have explored hybrid strategies to potentially minimize stent burden. The PEPCAD-CTO study evaluated the outcome of DCBs and DES used alongside BMS for CTO treatment. While similar rates of clinical outcomes were observed in the two groups, the DCB + BMS group required a shorter duration of DAPT and did not encounter late in-stent thrombosis [98]. A recent prospective, observational, multicenter study compared the outcomes of “less DES strategy” (DCB alone or combined with DES) and “DES-only strategy” in treating de novo coronary CTO: in the DCB group, the average stent length per lesion and LLL were significantly lower compared with the DES-only group. There were no significant differences in restenosis occurrence between the two groups [99]. A retrospective study also tested a DCB-only strategy and a hybrid strategy (DCB in combination with DES), showing that the recanalization results and long-term outcomes are comparable between the two groups [100].

Although clinical data are limited, preliminary evidence suggests a synergistic hybrid treatment strategy combining DCB and DES. This approach reduces the overall stent length while preserving the stents’ scaffolding properties where necessary. Additionally, DEBs facilitate natural dilation of the vessel post-PCI, addressing a major limitation of stenting in CTO lesions [95].

### 3.6. Acute Coronary Syndromes

Acute coronary syndromes (ACS) carry a heightened risk of thrombosis and PCI complications. Diffuse vasospasm and thrombus presence can lead to distal embolization, causing the no-reflow phenomenon and frequent vessel undersizing and strut malapposition. Thus, deferred stenting is often preferred over immediate deployment in primary PCI lesions, particularly with a high thrombus burden. DCB treatment requires similar techniques, but care must be taken to avoid using DCBs in cases with clear angiographic thrombus, as it may impede drug delivery to the vessel wall. Small-scale studies have explored DCB use in both STEMI and NSTEMI patients.

#### 3.6.1. STEMI Population

The REVELATION trial for STEMI, which was a small, prospective, randomized, and single-center, involved 120 patients with a new, not heavily calcified culprit lesion in a native coronary artery and a residual stenosis of less than 50% following pre-dilatation. These patients were randomized to receive either a DCB or a DES. The primary endpoint was fractional flow reserve at 9 months. At the two-year follow-up, the clinical outcomes for both the DCB and DES groups were similar [101].

Another study performed in STEMI patients by Gobic et al. also showed similar results at 6-month FU. Seventy-five patients with STEMI were randomized 1:1 to DES and DCB. The study focused on MACE and LLL within 6 months following primary PCI. At the 6-month mark, reinfarction occurred in 5.4% of the DES group and 5.3% of the DCB group (RR = 1.03, 95% CI [0.15–6.91], *p* = 0.98). The DES group experienced a LLL of 0.10 ± 0.19 mm, while the DCB group saw a change of −0.09 ± 0.09 mm (*p* < 0.05) [102].

In the recent DEB-AMI trial, 150 STEMI patients were randomly assigned to one of three groups: A (BMS), B (DCB, combined with BMS), and C (DES) following successful thrombus aspiration. The primary endpoint was the angiographic late stent lumen loss at 6 months. Out of the 150 randomized patients, 96.7% underwent a successful procedure. The late lumen loss was 0.74 ± 0.57 mm in group A, 0.64 ± 0.56 mm in group B, and 0.21 ± 0.32 mm in group C (*p* = 0.01). The rates of binary restenosis were 26.2% for group A, 28.6% for group B, and 4.7% for group C (*p* = 0.01). The incidence of MACE was 23.5% in group A, 20.0% in group B, and 4.1% in group C (*p* = 0.02) [103].

One hundred patients presenting with STEMI were prospectively enrolled in the PAPPA (PAclitaxel-eluting balloon angioplasty in Primary Percutaneous coronary intervention in Amsterdam) pilot study. The patients underwent primary PCI with DCB, with additional stenting permitted only in instances of type C to F coronary dissection or residual stenosis exceeding 50%. The primary endpoint encompassed a composite of cardiac death, recurrent myocardial infarction, and TLR. Out of the cohort, 59 patients received DCB angioplasty alone, while 41 required supplementary stenting. The one-year clinical follow-up was completed for 98 patients, during which five serious adverse cardiac events were reported (5%). Cardiac death was observed in two patients, and three patients underwent TLR [104].

#### 3.6.2. NSTEMI Population

In the PEPCAD NSTEMI trial, 210 patients were randomized to compare DCB with primary stenting (BMS or DES). The main inclusion criterion was the presence of a culprit lesion without significant angiographic evidence of a large thrombus. Over an average follow-up of 9.2 months, DCB treatment proved to be non-inferior to stenting, with a 3.8% incidence of TLF (primary endpoint) compared to 6.6% in the intention to treat analysis (*p* = 0.53). No significant difference was observed between BMS and second-generation DES. MACE rate was 6.7% in the DCB group vs. 14.2% in the DES group (*p* = 0.11), and 5.9% versus 14.4% in the per-protocol analysis (*p* = 0.056), respectively [105].

Recently, a meta-analysis of three randomized trials and one observational study comparing DCB vs. DES treatment for AMI, showed a similar rate of MACE, all-cause mortality, cardiac death, myocardial infarction, and TLR between the two groups at 9 months.

Additionally, LLL with DCBs was similar to that observed with stenting [106].

In summary, it is advisable to exercise caution when utilizing DCBs in this context, pending the results of large-scale studies involving ACS patients.

### 3.7. High Bleeding Risk and Diabetes Mellitus

Bleeding after PCI is associated with increased morbidity and mortality, and it remains a significant clinical challenge due to the occurrence of severe bleeding especially in patients with a high burden of comorbidities who take DAPT during the first year [107,108]. Consequently, it is essential to meticulously plan an optimal treatment strategy. for HBR patients regarding both revascularization and antithrombotic medication. In the last few years, many efforts have been made to define the ideal length of DAPT in HBR patients after PCI. With many of the current-generation DES, reduced DAPT can be prescribed in case of clinical need [61]. Current-generation DES are safer than the previous ones but are still associated with a risk of thrombotic events. Notably, most (~65%) of them occur during the first 30 days after revascularization [109,110].

DCB treatment was proposed in place of stenting in patients with HBR. Definitive guidelines for the duration of antiplatelet therapy following DCB-only PCI remain unavailable. According to the ESC Guidelines, a 6-month duration of DAPT is recommended for patients receiving DCB for ISR, without distinguishing between DES and DCB-only PCI. Nonetheless, at least 30 days of DAPT has consistently been implemented with DCB-only PCI, as its safety was demonstrated in retrospective studies and in two RCTs evaluating DCB PCI in de novo lesions [68,111,112]. Indeed, DCB was shown to be non-inferior to DES in small native coronary artery disease in MACE up to 12 months of follow-up in the BASKET-SMALL-2 trial [113] and superior to BMS in HBR patients in the DEBUT trial [65]. Therefore, the international DCB Consensus group recommends 1-month DAPT after elective PCI with DCB [4].

Interestingly, some studies have focused on the possibility of single antiplatelet therapy (SAPT) after DCB PCI in HBR patients. In a retrospective study, Rasanen et al. proved that this approach proved successful in achieving a low incidence of TLR, MI, and mortality, with a 1-year MACE rate (4.7%) and cardiovascular death rate (2.9%) numerically lower than in randomized clinical trials (RCTs) testing DES in the HBR population. Furthermore, despite the elderly cohort having a high comorbidities burden, the 1-year risk of significant bleeding (Bleeding Classification System Definitions, BARC 2–5) was 10.5%, which is numerically lower compared to other PCI studies in HBR patients [114]. Cortese et al. retrospectively analyzed a cohort of patients who underwent PCI with DCB for de novo coronary lesions: after 1 year, patients treated with SAPT, because of HBR or active bleeding, and those treated with DAPT did not significantly differ about the primary endpoint of MACE and its components (cardiac death, MI and TLR), but a reduction in the cumulative rate of 2 to 5 BARC bleedings was observed in the SAPT group [110]. Diaz et al. demonstrated that implementing SAPT with a P2Y12 inhibitor following DCB PCI in HBR patients is both safe and effective for treating de novo coronary lesions in cases of stable coronary artery disease and ACS [115]. In addition, Mangier et al. later published a post hoc analysis from EASTBOURN registry to compare the SAPT and DAPT therapy following a PCI with an SCB for de novo lesions or ISR. Intriguingly, at the 12-month follow-up, no differences were found in terms of TLR and MACE between the two groups with a high rate of BARC 3–5 bleeding in the SAPT group (probably a reflection of the fact this patient’s group is more prone to bleeding) [116].

Although unequivocal data are still lacking regarding the shortening of DAPT, or the possibility of using SAPT in patients undergoing DCB PCI, the latter represents a promising strategy for peculiar settings such as HBR, allowing for a reduction in the rate of bleeding without compromising preventive efficacy on ischemic events [117]. In this regard, encouraging preliminary data on the efficacy and safety of a low-intensity antiplatelet approach among patients with ACS treated with PCBs have recently been presented (REC-CAGEFREE II study) [118]. At the 12-month follow-up, the primary endpoint (including all-cause death, myocardial infarction, stroke, revascularization, and major bleeding events) revealed that the stepwise de-escalation approach was not inferior to the standard regimen.

Another setting of interest that has emerged as possible target for DCBs is diabetes mellitus (DM). Patients with DM typically have a diffuse, long and multivessel disease, and suffer from worse clinical outcomes after PCI with stent implantation: in particular, they are at higher risk of restenosis, stent thrombosis and MI because of more challenging coronary anatomies, and more stents of longer lengths and smaller diameters are usually required [119,120]. Therefore, these patients might receive a benefit from a minimalistic approach based on DCB therapy. Li et al. conducted a meta-analysis and aimed to explore the comparative short-term efficacy and safety of DCB vs. DES for treating small-vessel coronary artery lesions in diabetic patients [121]. They included six RCTs with 847 patients showing that DCB-PCI was associated with fewer MACE, MI, TLR, target vessel revascularization, BR and LLL [65,122,123,124,125,126]. On the other hand, success rate, death, MLD and net lumen gain were comparable to DES-PCI. Encouraging results also emerged from studies that tested DCB in diabetic patients undergoing PCI for in-stent restenosis or de novo lesions [127], as well as in the setting of multivessel coronary artery disease [128]. In the latter setting, the clinical benefit of DCB treatment for de novo multivessel CAD appears to be higher in diabetic patients. Indeed DCB-PCI significantly reduced the rate of adverse cardiovascular events compared with DES-PCI in diabetic patients, whereas no significant difference in clinical outcomes between DCB-PCI and DES-PCI was observed in non-diabetic patients [128].

Interestingly, utilizing DCBs may mitigate the prognostic disadvantages, such as a higher risk of cardiac events and repeated revascularization, in diabetic patients compared to non-diabetic patients. In the EASTBOURNE DIABETES registry, diabetic patients treated with SCB did not exhibit a significant increase in TLR compared to their non-diabetic counterparts [129].

All the available data suggest that DCB may be an effective and safe revascularization strategy for patients with DM. However, further studies are needed to validate these promising expectations and investigate the long-term efficacy and safety.

## 4. Future Perspectives

Despite the evidence accrued over the years supporting DCBs, their use in clinical practice is still limited. Currently, in Europe, the European Society of Cardiology guidelines recommend the use of DCBs solely for the treatment of In-Stent ISR [55]. On the other hand, in the United States, the Food and Drug Administration (FDA) has only recently approved the use of Paclitaxel-coated balloons for ISR treatment, with the AGENT balloon (Boston Scientific, USA) being the first to receive such approval. Nevertheless, in Europe, DCBs are employed in clinical practice in numerous clinical contexts, especially for the treatment of de novo lesions in small vessels or in the presence of diffuse disease. In these clinical scenarios, a gentle pre-treatment of the lesion followed by DCBs allows for the implementation of the “leaving nothing behind” strategy, thereby reducing the risks of device failure associated with DES implantation. This approach leaves the opportunity to adopt a hybrid approach with stent implantation only when necessary as bail-out.

On the one hand, research must continue to investigate the use of DCBs in various contexts, on the other hand, given the significant differences among the various balloons, it must also investigate if a technology is superior to another and, if so, in which setting.

Actually, there are no randomized studies comparing Sirolimus- and Paclitaxel-coated balloons and in the future, research will certainly move in this direction.

## 5. Conclusions

Although DES represent the most prevalent and well-established therapeutic approach in contemporary PCI, the utilization of DCBs is consistently on the rise across diverse clinical scenarios. In the near future, DCBs may play an increasingly significant role, representing a viable alternative to the current gold standard. Certainly, further evidence is needed to understand the ideal context for the use of DCBs and, above all, the differences between the various devices currently available on the market.

## Figures and Tables

**Table 1:** Commercially available coronary DCBs.

Device	Drug	Dose (μg/mm^2^)	Productor	Approval
Agent	Paclitaxel	2	Boston Scientific	CE certified, FDA approved
Elutax SV	Paclitaxel	2.2	Aachen Resonance	CE certified
SeQuent Please	Paclitaxel	3	BBraun	CE certified
Pantera Lux	Paclitaxel	3	Biotronik	CE certified
Danubio	Paclitaxel	2.5	Minvasys	CE certified
Dior II	Paclitaxel	3	Eurocor	CE certified
RESTORE	Paclitaxel	3	Cardionovum	CE certified
IN.PACT Falcon	Paclitaxel	3.5	Medtronic	CE certified
AngioSculpt X	Paclitaxel	3	Spectranetics	CE certified
Essential	Paclitaxel	3	iVascular	CE certified
Chocolate Touch	Paclitaxel	3	QT Vascular	CE certified
Selution	Sirolimus	1	Med Alliance	CE certified
SeQuent Please SCB	Sirolimus	4	BBraun	CE certified
Magic Touch	Sirolimus	1.27	Concept	CE certified
Mozec SEB	Sirolimus	3	Meril	CE certified
Biolimus A9 BCB	Biolimus A9	3	Biosensors International	-

CE: Conformité Européenne; DCB: drug-coated balloon; FDA: U.S. Food and Drug Administration.

**Table 2:** Clinical trials on the use of DCB for in-stent restenosis.

STUDY	Design	Follow-Up	Endpoints	*p*-Value
**BMS-ISR**				
PACCOCATH ISR-I	PCB (n = 26) vs. POBA (n = 26)	Angiographical at 6 month and clinical at 12 month	LLL: 0.03 ± 0.48 mm vs.0.74 ± 0.86 mm	**0.002**
			MACE: 4% vs. 31%	**0.01**
PACCOCATH ISR-II	PCB (n = 54) vs. POBA (n = 54)	Angiographical at 6–9 months and clinical at 24 months	LLL: 0.11 ± 0.45 mm vs.0.81 ± 0.79 mm	**0.001**
			MACE: 27.8% vs. 59.3%	**0.009**
PEPCAD II	PCB (n = 66) vs. PES (n = 65)	Angiographical at 6 month and clinical at 12 month	LLL: 0.17 ± 0.42 mm vs. 0.38 ± 0.61 mm	**0.03**
			BR%: 7% vs. 20%	0.06
			MACE: 9% vs. 22%	0.08
SEDUCE	PCB (n = 25) vs. EES (n = 25)	Angiographical at 9 month and clinical at 1–8–12 months	LLL: 0.28 mm vs. 0.07 mm	0.1
LLL: 0.28 mm vs. 0.07 mm	PCB (n = 95) vs. EES (n = 94)	Angiographical at 6–9 months and clinical at 12 months	LLL: 0.14 ± 0.5 mm vs. 0.04 ± 0.5 mm; binary restenosis: 9.5% vs. 4.7%	0.22
			MACE: 8% vs. 6%	0.60
TIPS	PCB (n = 68) vs. EES (n = 68)	Angiographical at 12 ± 2 months and clinical at 6–12 months	LLL: 0.02 mm vs. 0.19 mm	**0.0004**
			MACE: 10.29% vs. 19.12%	0.213
			Binary restenosis: 8.7% vs. 19.12%	0.078
**DES-ISR**				
RIBS IV	DCB (n = 154) vs. EES (n = 155)	Angiographical at 6–9 months and clinical at 12–24–36 months	MLD: 1.80 ± 0.6 mm vs. 2.03 ± 0.7 mm	**<0.01**
			MACE: 12.3% vs. 20.1%	**0.04**
ISAR-DESIRE 3	PCB (n = 137) vs. PES (n = 131) vs. POBA (n = 134)	Angiographical at 6–8 months and clinical at 1–12 months	Binary restenosis: 38% vs. 37.4% vs. 54.1%	**0.007**
			TLR: 22.1% vs. 13.5% vs. 43.5%	*p* (PCB vs. PES) = 0.09, *p* (PCB vs. POBA) < 0.001
PEPCAD CHINA ISR	PCB (n = 110) vs. PES (n = 110)	Angiographical at 9 months and clinical at 1–6–9–12 months	LLL: 0.46 ± 0.51 mm vs. 0.55 ± 0.61 mm	**0.0005**
			TLR: 14.5% vs. 13.6%	0.84
ISAR DESIRE IV	PCB (n = 127) vs. SCB + PCB (n = 125)	Angiographical at 6–8 months and clinical at 12 months	In-segment DS%: 35.0 ± 16.8% vs. 40.4 ± 21.4%	**0.047**
			TLR: 21.8% vs. 16.2%	0.26
			MACE: 23.3% vs. 18.4%	0.35
RESTORE	DCB (n = 86) vs. EES (n = 86)	Angiographical at 9 months and clinical at 12 months	LLL: 0.15 ± 0.49 mm vs. 0.19 ± 0.41 mm	0.54
			In-segment MLD: 1.80 ± 0.69 mm vs. 2.09 ± 0.46 mm	**0.03**
			In-stent MLD: 1.90 ± 0.71 mm vs. 2.29 ± 0.48 mm	**0.005**
			In-segment DS%: 34% ± 21% vs. 26% ± 15%	**0.05**
			In-stent DS%: 33% ± 21% vs. 21% ± 15%	**0.002**
			MACE: 7.0% vs. 4.7%	0.51
PEPCAD-DES	PCB (n = 72) vs. POBA (n = 38)	Angiographical at 6 months and clinical at 6 months	LLL: 0.43 ± 0.61 mm vs. 1.03 ± 0.77 mm	**<0.001**
			MACE: 20.8% vs. 52.6%	**0.001**
**MIXED ISR**				
BIOLUX	DCB (n = 157) vs. DES (n = 72)	Angiographical at 6 months and clinical at 6–12–18 months	LLL: 0.03 ± 0.40 mm vs. 0.20 ± 0.70 mm	0.40
			TLF: 16.7% vs. 14.2%	0.65
DARE	DCB (n = 141) vs. DES (n = 137)	Angiographical at 6 months and clinical at 12	MLD: 1.71 ± 0.51 mm vs. 1.74 ± 0.61 mm	**<0.0001**
			TVR: 7.1% vs. 8.8%	0.65

DCB, drug-coated balloon; BMS, bare-metal stent; ISR, in-stent restenosis; DES, drug-eluting stent; EES, everolimus-eluting stent; PES, paclitaxel-eluting stent; PCB, paclitaxel-coated balloon; SCB, sirolimus-coated balloon; TLF, target lesion failure; TLR, target lesion revascularization; TVR, target vessel revascularization; POBA, plain old balloon angioplasty; MLD, minimum lumen diameter; MACE, major advance cardiovascular event; LLL, late lumen loss; DS%, percent diameter stenosis; BR%, Binary Restenosis Rate.

**Table 3:** Clinical trials on the use of DCB for bifurcation lesions.

STUDY	Design	Follow-Up	Endpoints	*p*-Value
**DCB in SB; DES/BMS in MB**				
DCB Bifurcation Study	DES in MB, PCB in SB (n = 50) vs. DES in MB, POBA (n = 50) in SB	Angiographical and IVUS at 12 month and clinical at 12 month	LLL: 0.09 ± 0.4 mm vs. 0.40 ± 0.5 mm	**0.01**
			MACE: 11% vs. 24%	0.11
			TLR: 12% vs. 22%	0.16
BABILON	BMS in MB, PCB in SB (n = 52) vs. DES in MB (n = 56)	Angiographical at 9 months and clinical at 1–6–12–24 months	LLL: in MB: 0.31 ± 0.48 mm vs. 0.16 ± 0.38 mm	0.15
			In SB: −0.03 ± 0.51 mm vs. 0.04 ± 0.76 mm	0.983
			MACE: 17.3% vs. 7.1%	0.105
			TLR: 15.4% vs. 3.6%	**0.045**
BEYOND	DES in MB, PCB in SB (n = 113) vs. DES in MB, POBA (n = 109) in SB	Angiographical at 9 months and clinical at 1–6–9 months	LLL: 0.06 mm ± 0.32 mm vs. 0.18 mm ± 0.34 mm	**<0.0001**
			Restenosis rate: 28.7% vs. 40%	**<0.0001**
			MACE: 0.9% vs. 3.7%	0.16
			Non-fatal acute myocardial infarction: 0% vs. 0.9%	0.49
**DCB-only in SB**				
PEPCAD-BIF	DCB (n = 32) vs. POBA (n = 32)	Angiographical at 9 months	LLL: 0.13 mm vs. 0.51 mm	**0.013**
			Restenosis rate: 6% vs. 26	**0.045**

DCB, drug-coated balloon; BMS, bare-metal stent; DES, drug-eluting stent; PCB, paclitaxel-coated balloon; TLR, target lesion revascularization; POBA, plain old balloon angioplasty; MACE, major advance cardiovascular event; LLL, late lumen loss; MB, main branch; SB, side branch.
